# Selective defoliation affects plant growth, fruit transcriptional ripening program and flavonoid metabolism in grapevine

**DOI:** 10.1186/1471-2229-13-30

**Published:** 2013-02-22

**Authors:** Chiara Pastore, Sara Zenoni, Marianna Fasoli, Mario Pezzotti, Giovanni Battista Tornielli, Ilaria Filippetti

**Affiliations:** 1 Department of Fruit Tree and Woody Plant Science, University of Bologna, Viale Fanin, 46, 40126, Bologna, Italy; 2 Department of Biotechnology, University of Verona, Strada Le Grazie 15, 37134, Verona, Italy

**Keywords:** *Vitis vinifera*, Defoliation, Berry ripening, Transcriptome, Flavonoid, Source-sink balance

## Abstract

**Background:**

The selective removal of grapevine leaves around berry clusters can improve the quality of ripening fruits by influencing parameters such as the berry sugar and anthocyanin content at harvest. The outcome depends strongly on the timing of defoliation, which influences the source–sink balance and the modified microclimate surrounding the berries. We removed the basal leaves from *Vitis vinifera* L. cv Sangiovese shoots at the pre-bloom and veraison stages, and investigated responses such as shoot growth, fruit morphology and composition compared to untreated controls. Moreover, we performed a genome-wide expression analysis to explore the impact of these defoliation treatments on berry transcriptome.

**Results:**

We found that pre-bloom defoliation improved berry quality traits such as sugar and anthocyanin content, whereas defoliation at veraison had a detrimental effect, e.g. less anthocyanin and higher incidence of sunburn damage. Genome-wide expression analysis during berry ripening revealed that defoliation at either stage resulted in major transcriptome reprogramming, which slightly delayed the onset of ripening. However, a closer investigation of individual gene expression profiles identified genes that were specifically modulated by defoliation at each stage, reflecting the uncoupling of metabolic processes such as flavonoid biosynthesis, cell wall and stress metabolism, from the general ripening program.

**Conclusions:**

The specific transcriptional modifications we observed following defoliation at different time points allow the identification of the developmental or metabolic processes affected in berries thus deepening the knowledge of the mechanisms by which these agronomical practices impact the final berry ripening traits.

## Background

Grapevine canopy management is used to control the microclimate around the berry clusters to optimize fruit composition and health, retaining at the same time an optimal leaf-area-to-fruit ratio in vines. The microclimate influences many berry quality traits, e.g. sunlight increases the level of sugars, anthocyanins and phenolics [1-3]. The precise response is dependent on the grapevine genotype [4-7] and also on the berry temperature [8-12].

Berry cluster microclimate is affected by several viticultural practices such as the training system, row orientation, leaf canopy density and the position of the cluster. Removing leaves around the clusters is a powerful and widely-used strategy to control berry illumination and temperature, although the impact depends on the timing of treatment. Thus, defoliation can be implemented at any time between pre-bloom and veraison, with different consequences. The photosynthetic activity of basal leaves at veraison is lower than that of intermediate and apical leaves, so defoliation at this stage has a strong impact on light and temperature exposure but a limited impact on the source–sink balance. In contrast, the removal of basal leaves before flowering affects the source–sink balance significantly, reducing yields and improving berry quality in many cultivars and vineyard environments [13-16]. These responses reflect the strict association between berry set and the number of source leaves on the shoot during flowering [17]. Removing basal leaves reduces the berry set percentage and growth rate during the green stage, thus reducing yields and allowing the re-growth of leaves on lateral shoots during the same vegetative season, resulting in a complex realignment of growth and physiological parameters [18]. Flavonoid biosynthesis is one process most impaired by defoliation in berries but the outcome seems dependent on the timing of defoliation and on the genotype [6,7,19].

To date the knowledge regarding the physiological responses of grape subjected to canopy management practices are mainly restricted to plant growth responses and fruit ripening parameters. We have recently approached the study of cluster thinning in grapevine through a genome wide transcriptomic profiling which, integrated with agronomic and biochemical data, provided insights into the molecular basis of berry ripening induced by this vineyard management technique [20].

In this work we compared the impact of defoliation before flowering and at veraison on Sangiovese berry quality traits, focusing on associated changes in the transcriptome. We integrated global gene expression profiles and agronomic and biochemical data, allowing us to determine the molecular mechanisms underlying changes in berry composition caused by defoliation. The comparison of gene expression profiles in defoliated and control vines revealed a transcriptional response common to berries from both treatment programs at the end of veraison, but also a more extensive transcriptome rearrangement in berries defoliated before flowering that may explain the specific biochemical and structural changes in these berries.

## Results and Discussion

### The impact of defoliation on berry skin temperature

**Figure 1:**
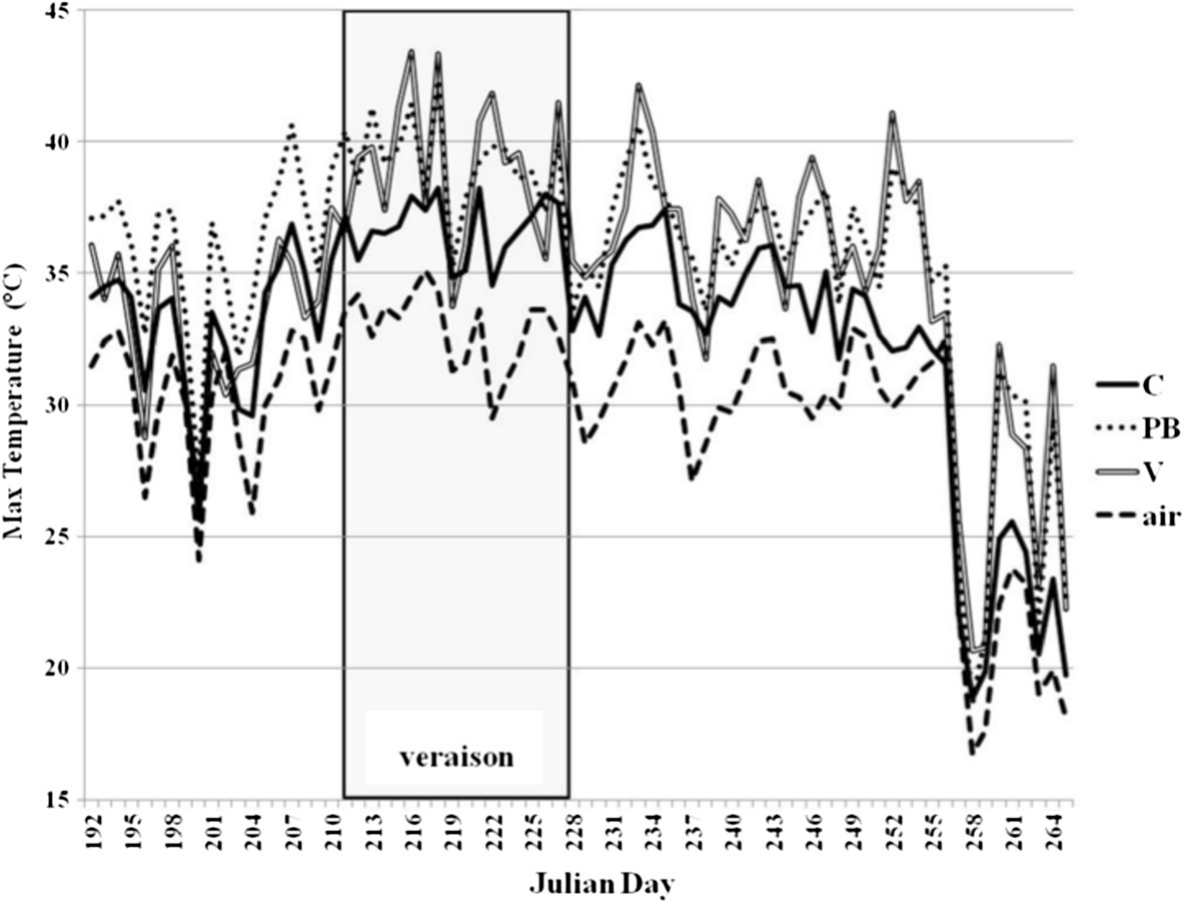
**Trend in maximum diurnal temperature from bunch closure to harvest in C, PB and V Sangiovese berries.** Maximum air temperature is also shown. Gray background represents the phase of veraison.

We monitored the berry skin temperature and surrounding air temperature from bunch closure to harvest in the C (untreated control), PB (pre-bloom defoliation) and V (veraison defoliation) treatment groups, noting that the highest maximum skin temperature was achieved during veraison, JD 211–227 (Figure 1). The maximum skin temperature in the PB berries was higher over the monitoring period in comparison to C, while the temperature in the V berries increased rapidly compared to C only after the treatment, reaching and sometimes exceeding the maximum values observed in the PB berries (Figure 1). Many metabolic processes slow down or stop at higher temperatures [21], and the temperature threshold in grapevine above which anthocyanin synthesis begins to decline is thought to be 30°C [4,11,22]. From veraison to harvest, berries in the C sample were exposed to temperatures >30°C for less time (<270 h) than the PB and V samples (~300 h).

### The impact of defoliation on agronomic and ripening parameters

All the sampled vines showed a uniform leaf area before blooming (Table 1). Despite the removal of ~70% of basal leaves from the PB vines, there was no significant difference in leaf area between the PB and C vines at harvest. More lateral shoots appeared on the PB vines, explaining the recovery in leaf area after defoliation despite the loss of main leaves. However, there was a significant reduction in total leaf area at harvest in the V vines because there was no leaf regrowth after veraison (Table 1). The yield per vine at harvest in the PB vines was 30% lower than C vines and 20% lower than V vines although the total number of clusters was not affected (Table 2). This indicates that a major impact of PB defoliation was a reduction in cluster weight reflecting the lower berry number (29% less than C and 18% less than V). Compared to the control treatment, pre-bloom defoliation did not significantly affect berry size during ripening but the clusters were less compact (Table 2 and Additional file 1).

**Table 1:** Influence of pre-bloom (PB) and veraison defoliation (V) on leaf area components of Sangiovese grapevines compared to a non-defoliated control (C)

	***C***	***PB***	***V***
Total leaf area/vine (m^2^) before pre-bloom defoliation	1.7	1.69	1.75
Leaf area/vine (m^2^) removed at pre-bloom defoliation	0	1.16	0
Leaf area/vine (m^2^) left after pre-bloom defoliation	1.70 a	0.53 b	1.75 a
Leaf area/vine (m^2^) removed at véraison defoliation	0	0	1.4
Final main leaf area/vine (m^2^)	2.52 a	1.84 b	1.72 b
Final laterals leaf area/vine (m^2^)	1.21 b	1.42 a	1.11 b
Total final leaf area/vine (m^2^)	3.73 a	3.26 ab	2.83 b

Means within rows followed by different letters differ significantly at p < 0.05 according to the Student–Newman–Keuls test.

**Table 2:** Influence of pre-bloom (PB) and veraison defoliation (V) on agronomic parameters, ripening parameters, and the flavonol and anthocyanin content of Sangiovese berries compared to non-defoliated controls (C) at harvest

		***C***	***PB***	***V***
***Agronomic parameters***	Yield/vine (kg)	6.3 a	4.4 b	5.5 a
	Cluster number/vine	16	16	16
	Cluster weight (g)	394 a	280 b	343 a
	Berry weight (g)	2.37	2.26	2.33
	Berry number/cluster	166 a	124 b	147 a
	Cluster compactness (OIV rating)	7.5	6.8	7.8
	Bunch rot/cluster (%)	5	2.1	2.3
	Sunburn damage/cluster (%)	0.36 b	1.24 b	6.45 a
	Leaf area/yield (m^2^/kg)	0.6 b	0.8 a	0.5 b
	Berry skin thickness (μm)	232.3 b	255.6 a	223.8 b
***Ripening parameters***	°Brix	20.8 b	22.2 a	20.7 b
	TA (g/L)	7.6 a	6.8 b	6.7 b
	pH	3.38 b	3.45 a	3.45 a
***Flavonols***	Total flavonols (mg/g of skin)	0.32 b	0.71 a	0.67 a
	Quercetin (%)	79.5 b	83.3 a	84.5 a
	Myricetin (%)	17.7 a	11.4 b	10.5 b
	Kaempferol (%)	2.8 b	5.3 a	5.0 a
***Anthocyanins***	Total anthocyanins (mg/g of skin)	4.87 ab	5.35 a	4.33 b
	Delphinidin-3-G (%)	15	15.3	15.2
	Cyanidin-3-G (%)	28.7 b	32.8 ab	42.3 a
	Petunidin-3-G (%)	14.5	14	12.2
	Peonidin-3-G (%)	13.5	14.3	13.1
	Malvidin-3-G (%)	28.3 a	23.6 ab	17.2 b
	3^′^4^′^-OH anthocyanins (%)	42.2 b	47.1 ab	55.3 a
	3^′^4^′^5^′^-OH anthocyanins (%)	57.8 a	52.9 a	44.7 b

Means within rows followed by different letters differ significantly at p < 0.05 according to the Student–Newman–Keuls test.

The incidence of bunch rot was lower under both defoliation treatments (Table 2). The berry skin thickness was 13% higher in PB compared to C and V berries, as determined by texture analysis (Table 2). The impact of PB defoliation on berry size and cluster compactness can vary considerably across seasons, but the overall effect on yield is constant because of the strong relationship between carbohydrate supply before flowering and fruit-set [14,15,23]. Berry clusters in the V treatment group showed more sunburn damage than the other groups, probably reflecting the sudden increase in temperature and exposure to sunlight after defoliation (Table 2). In contrast, berries in the PB group were more resistant to sunburn probably due to their thicker skins [18].

The leaf-area-to-fruit ratio per vine in the PB treatment group was significantly higher than in the other groups, resulting in the accumulation of more soluble solids in the berries and thus a differential of ~2 °Brix (Table 2; Additional file 1). Despite this, there were no significant differences among the three treatment groups in the sugar accumulation profile, except a delay in the PB berries at the end of veraison (225–228 JD) which was overcome during the last phase of ripening (Additional file 1). Sugar accumulation during ripening may be influenced by the dynamic changes in canopy leaf age and photosynthesis induced by pre-bloom defoliation, as previously observed in Sangiovese vines where photosynthesis in the defoliated shoots was reduced significantly from defoliation until full veraison and exceeded the control level only after veraison [16].

The titratable acidity was lower in PB and V compared to C berries (Table 2 and Additional file 1). Although we did not measure the individual malic and tartaric acid fractions, the total acidity is probably linked to the higher berry temperature, which is responsible for accelerating the breakdown of malic acid [24] as previously reported [15,16,25].

### The impact of defoliation on flavonol and anthocyanin accumulation

**Figure 2:**
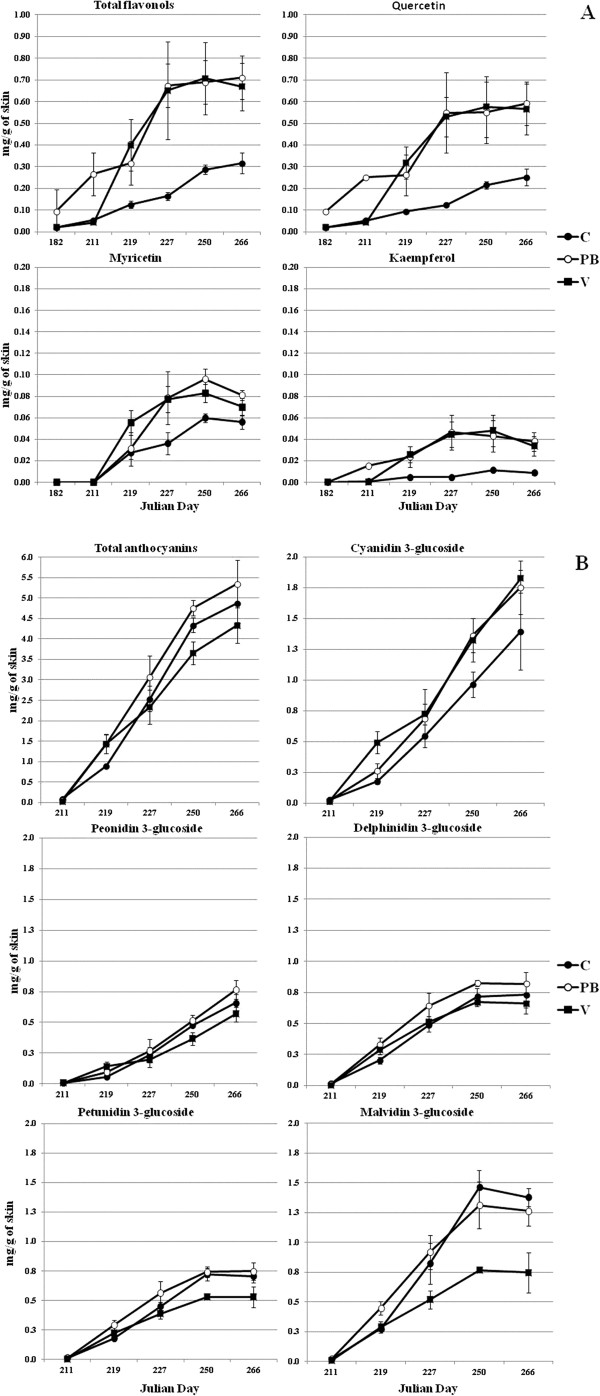
**Effects of pre-bloom (PB) and veraison (V) defoliation on the accumulation of flavonols (A) and anthocyanins (B) in the skin of Sangiovese berries compared to a non-defoliated control (C).** Vertical bars indicate the standard deviation of the mean (n = 3 biological replicates).

Total flavonol levels were significantly higher in PB compared to C and V berry skins at the beginning of veraison, but there was no difference at harvest between PB and V (Figure 2A, Table 2). Both defoliation treatments, in fact, induced flavonol biosynthesis to a similar extent, resulting in up to 0.71 mg of total flavonols per gram of skin in PB berries compared to 0.32 mg/g in C berries. Sunlight is known to enhance flavonol accumulation in berries [26] and there is a strong positive correlation between illumination and flavonol levels reflecting their role as UV protectants [11,27]. Temperature is thought to have little or no impact on berry flavonol biosynthesis [11,27,28]. In Cabernet Sauvignon berries, the impact of defoliation on flavonol biosynthesis through increased light exposure appears to reflect the expression of two isoforms of flavonol synthase (FLS) [6].

The accumulation of individual flavonols was similar in PB and V berries. Quercetin is the main flavonol present in red grapes [29] and its concentration at harvest in PB and V berries was nearly twice that in C berries (Figure 2A). The accumulation profile of kaempferol, which usually accounts for ~5% of total flavonols [29], was similar to that of quercetin (Figure 2A). Defoliation only slightly affected the concentration of myricetin at harvest (Figure 2A). As a consequence PB and V berries accumulated relatively more quercetin and kaempferol than myricetin compared to C berries (Table 2). The shift in flavonol composition may be unique to Sangiovese berries because studies in other cultivars have shown that the abundance of all flavonol compounds increases following defoliation [11].

The total anthocyanin concentration in the berry skin at harvest was significantly higher in PB berries (5.35 mg/g) compared to V berries (4.33 mg/g) whereas C berries showed an intermediate value (4.87 mg/g) that did not differ significantly from either defoliation treatment (Table 2). However, the anthocyanin accumulation profile during ripening was clearly enhanced in PB berries and reduced in V berries compared to C (Figure 2B). These results may reflect the association between the source–sink balance in PB vines and the higher sugar concentration at harvest, which should positively affect anthocyanin accumulation [30]. Early leaf removal also induces skin thickening in PB berries, providing more epidermal layers for the storage of anthocyanin compounds. In V berries, the impact of leaf removal combined with the highest air temperature during the season may have inhibited anthocyanin synthesis and promoted anthocyanin degradation.

We also compared the abundance of the five monoglucoside anthocyanins in the different treatment groups (Figure 2B). Minor differences were detected in the levels of individual anthocyanins in PB and C berry skins at harvest, i.e. slightly higher levels of 3^′^4^′^-OH anthocyanins in PB berries (Table 2). The final 3^′^4^′^-OH/3^′^4^′^5^′^-OH anthocyanin ratio was higher in V berries reflecting the greater accumulation of cyanidin and the lower accumulation of malvidin during ripening (Table 2; Figure 2B). The relationship between anthocyanin composition and veraison defoliation appears to be cultivar dependent, since this treatment promotes a general increase in anthocyanin accumulation in Cabernet Sauvignon berries [6], but a variety-dependent increase in selected anthocyanins in Sangiovese (Table 2), Barbera [19] and Merlot berries [7].

### The impact of defoliation on the veraison berry transcriptome

**Figure 3:**
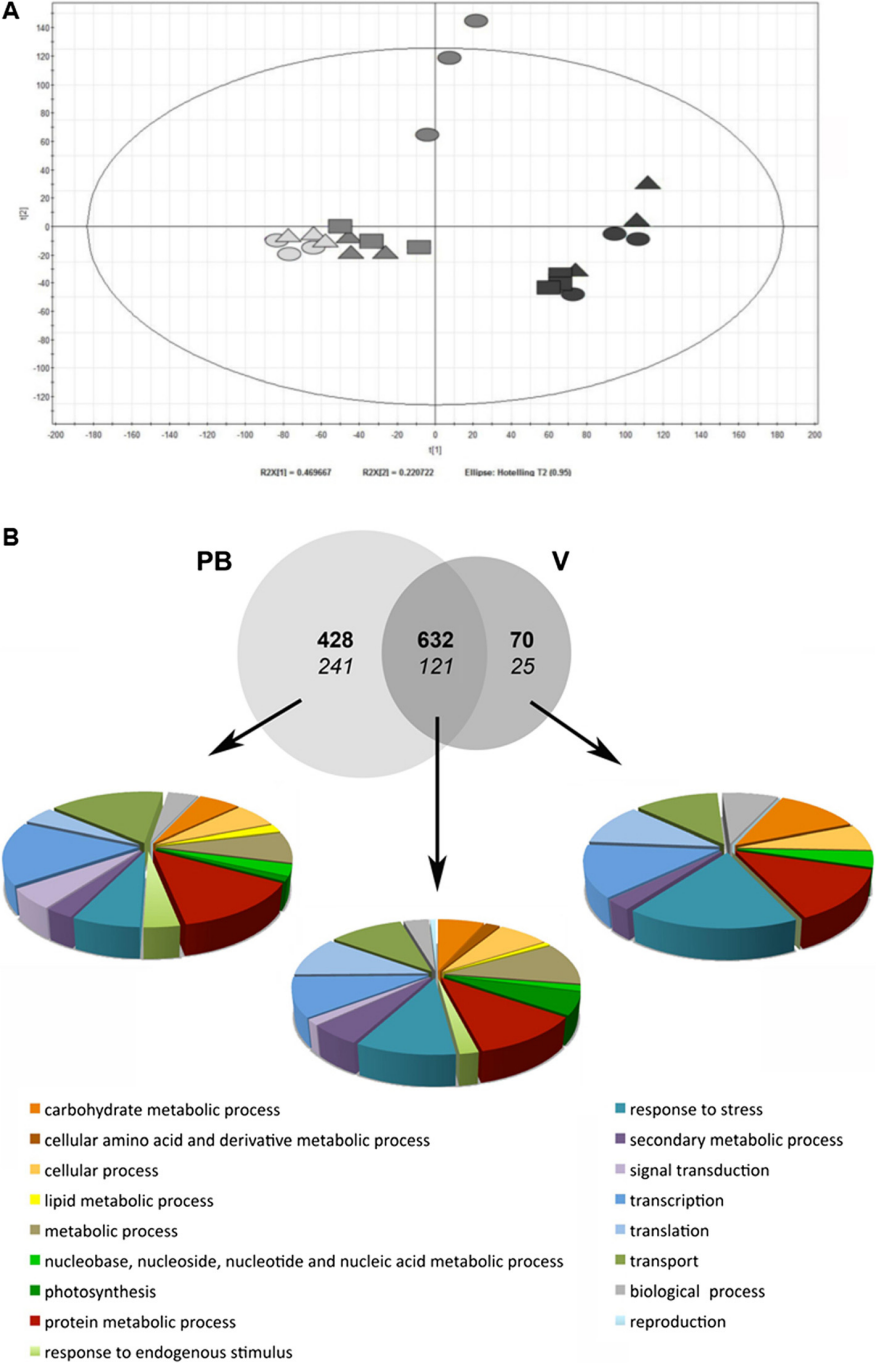
**Global transcriptome analysis of PB, V and C Sangiovese berries. **(**A**) Principal component analysis of all samples. Ovals represent C, triangles PB and rectangles V samples. The light gray corresponds to the BV, the gray to the EV and the black to the H stage. (**B**) Venn diagram summary of differentially-expressed genes in PB, V and C berries during ripening. Transcripts are divided into three different datasets according to their specific (PB or V) or common differential expression compared to C. Numbers in bold are referred to the up-regulated genes, while number in italic are referred to the down-regulated ones. Genes belonging to each dataset have been classified in Functional Categories.

To investigate the molecular changes that take place in response to defoliation, we compared the transcriptomes of (i) PB and C berries at time points JD 211, 227 and 266, corresponding to the beginning of veraison (BV), the end of veraison (EV) and harvest (H); and (ii) V and C berries at JD 227 and 266. Principal component analysis (PCA) of the global transcriptomic data revealed enough uniformity among the three biological replicates of each treatment to define associations between treatments (Figure 3A).

The two principal components, explaining ~70% of the overall variation, allowed us to clearly separate C from PB and V berries at the EV stage. However, there was a less distinct separation between PB and C berries at the BV stage, and between C berries and the other two treatments at harvest, suggesting that the major transcriptomic changes were induced by both defoliation treatments only at the EV stage. We also noted only minor transcriptomic differences between the BV to EV stages in the PB and V berries compared to C, suggesting that both defoliation treatments delayed the onset of ripening at the transcriptomic level.

To identify the most relevant differentially-expressed genes, we compared the C, PB and V berry transcriptomes at each time point using a Significance Analysis of Microarrays (SAM) unpaired comparison with a false discovery rate of 2%. No genes were differentially expressed with a fold change ≥2 between C and PB berries at the BV stage, suggesting that early defoliation has no impact on the berry transcriptome at this stage. However, at the EV stage we identified 1422 genes differentially expressed between C and PB berries, and 848 differentially expressed between C and V berries (Additional file 2). At harvest we identified four genes differentially expressed between C and PB berries and none differentially expressed between C and V berries. These data indicate that the C and PB berry transcriptomes are indistinguishable at the BV stage but begin to diversify at the EV stage followed by minimal additional changes at harvest, as anticipated by PCA.

At the EV stage, we found 753 genes that were differentially expressed in both of the defoliation treatments, 669 that differed only between C and PB berries, and 95 that differed only between C and V berries. All the transcripts were annotated against V1 of the 12X draft annotation of the grapevine genome allowing ~80% of the modulated genes to be identified (Additional file 2). The genes were distributed into 17 Gene Ontology (GO) functional categories and the percentage of genes representing each category was determined for each of the three data sets (Figure 3B).

The 753 genes differentially expressed in both of the defoliation treatments represented many different functional categories, predominantly “Protein metabolism”, “Response to stress”, “Transport”, “Transcription” and “Translation” but also “Photosynthesis” and “Secondary metabolism”, suggesting that defoliation generally affects many genes involved in berry ripening. The 669 PB-specific differentially-expressed genes predominantly represented “Transport”, “Transcription” and “Protein metabolism” and to a lesser extent “Response to stress”, “Translation” and “Photosynthesis”, but the “Signal transduction” and “Response to endogenous stimulus” categories also appeared to be significant. The 95 V-specific differentially-expressed genes predominantly represented the “Response to Stress” category, indicating that late defoliation induces berry stress. The “Transport”, “Transcription”, “Translation” and in particular “Carbohydrate metabolism” category were also significantly represented but there were no genes representing “Photosynthesis”, “Signal transduction” and “Response to endogenous stimulus”.

### Common impact of defoliation on the berry transcriptome

Among the 753 EV genes differentially expressed in both of the defoliation treatments, those with a fold change ≥5 were highly ranked in both treatment groups suggesting the major transcriptomic changes occur regardless of the timing of defoliation (Table 3). Interestingly, almost all the genes with higher expression levels in the defoliation treatment groups were downregulated in the C berries between BV and EV rather than upregulated specifically by defoliation, and those with lower expression levels in the defoliation treatment groups similarly tended to be upregulated in the C berries, suggesting that defoliation affects ripening by generally delaying the entire process (Additional file 2). These genes are described hereafter as not downregulated (NDR) and not upregulated (NUR) respectively, to distinguish them from genes, which are genuinely modulated by defoliation. Similarly, PCA revealed only minor differences between the EV and BV stage transcriptomes for the PB and V berries, whereas there was a more significant difference between those stages for the C berries (Figure 3A). The similar impact (i.e. delayed transcriptional changes associated with ripening) at EV following the two different defoliation treatments probably reflects the reduction in leaf area and its knock-on effect on the dynamic leaf-area-to-fruit ratio during the season.

**Table 3:** Differentially-expressed genes (FC ≥ 5) at EV in the comparisons PB vs C and V vs C

	**EV**				***Trend in C***
***Gene ID***	**FC PB/C**	**FC V/C**	***Gene description***	***Functional category***	**EV/BV**
VIT_19s0014g02520	12.22	11.50	ribosomal protein S8, Chloroplast 30S	photosynthesis	↓
VIT_02s0012g01950	11.82	11.21	photosystem II protein D1	photosynthesis	↓
VIT_08s0056g00590	11.37	8.92	chloroplast envelope membrane protein [Vitis vinifera]	photosynthesis	↓
VIT_07s0191g00130	10.78	9.22	histone H3.2	transcription	↓
VIT_09s0002g08330	10.14	7.48	Photosystem I P700 apoprotein A2	photosynthesis	↓
VIT_18s0001g08390	9.76	10.24	no hit		↓
VIT_08s0040g00640	9.40	5.64	no hit		↓
VIT_06s0009g02070	9.39	8.05	photosystem I assembly protein Ycf4	photosynthesis	↓
VIT_09s0054g01450	9.21	8.50	no hit		↓
VIT_11s0016g02030	9.09	7.30	cytochrome f [Vitis vinifera]	photosynthesis	↓
VIT_13s0067g03310	9.02	7.25	ATP synthase CF1 alpha subunit	photosynthesis	↓
VIT_00s0246g00190	8.82	7.43	NADH-plastoquinone oxidoreductase subunit 4 [Vitis vinifera]	photosynthesis	↓
VIT_06s0009g02080	8.15	6.26	chloroplast envelope membrane protein	photosynthesis	↓
VIT_00s0246g00180	7.99	6.70	NADH-plastoquinone oxidoreductase subunit 4 [Vitis vinifera]	photosynthesis	↓
VIT_05s0077g00190	7.97	7.86	no hit		↓
VIT_16s0039g00380	7.56	6.55	ribosomal protein S14 30S	translation	↓
VIT_04s0044g02000	7.35	6.66	no hit		↓
VIT_18s0122g00440	7.30	7.95	no hit		↓
VIT_18s0001g14260	7.26	5.91	no hit		↓
VIT_19s0014g02410	7.18	8.17	L-ascorbate peroxidase 1, cytosolic (APX1)	response to stress	↓
VIT_12s0034g01910	7.14	6.67	Glutelin type-A 3	metabolic process	
VIT_10s0092g00760	7.12	6.19	Unknown protein		↓
VIT_19s0027g01950	7.11	5.48	CPL4 (C-TERMINAL DOMAIN PHOSPHATASE-LIKE 4)	cellular process	↓
VIT_12s0059g02430	7.06	5.73	chloroplast envelope membrane protein [Vitis vinifera]	photosynthesis	↓
VIT_09s0054g00320	7.03	5.66	mannosyl-oligosaccharide 1,2-alpha-mannosidase	carbohydrate metabolism	↓
VIT_00s0267g00060	7.03	7.28	no hit		↓
VIT_19s0093g00150	7.00	6.84	Glutathione S-transferase 25 GSTU25	response to stress	↓
VIT_04s0043g00200	6.98	5.83	PROTEIN PHOSPHATASE 2A SUBUNIT A3 PP2AA3	protein metabolism	↓
VIT_01s0010g02310	6.89	5.49	ribosomal protein L23 50S	translation	↓
VIT_00s0505g00050	6.82	4.34	no hit		↓
VIT_04s0079g00350	6.73	6.74	tubulin, Beta	cellular process	↓
VIT_11s0016g05680	6.70	7.39	RAB GTPase activator	transport	↓
VIT_12s0034g01890	6.69	6.63	Cupin region	response to stress	
VIT_07s0005g03360	6.60	7.58	malate dehydrogenase, cytosolic	carbohydrate metabolism	↓
VIT_12s0034g01950	6.58	5.63	legumin	carbohydrate metabolism	
VIT_00s1707g00010	6.41	5.76	ribosomal protein S8, Chloroplast 30S	translation	↓
VIT_04s0008g01550	6.38	6.88	small molecular heat shock protein 17.5	response to stress	
VIT_13s0019g01440	6.35	6.46	no hit		↓
VIT_14s0030g00830	6.32	6.79	Superoxide dismutase	response to stress	↓
VIT_15s0045g00050	6.29	5.78	protein phosphatase 2A 65 kDa regulatory subunit	protein metabolism	↓
VIT_09s0018g00820	6.23	5.93	photosystem I assembly protein Ycf3	photosynthesis	↓
VIT_13s0047g00010	6.23	6.08	ZIFL2 (ZINC INDUCED FACILITATOR-LIKE 1)	transport	↓
VIT_11s0052g01680	6.16	5.71	photosystem II protein D1	photosynthesis	↓
VIT_19s0015g02700	6.11	5.45	Glutathione S-transferase 25 GSTU25	response to stress	↓
VIT_04s0044g01820	6.08	5.18	no hit		↓
VIT_13s0019g02760	6.02	6.28	heat shock protein 16.9 kDa class I	response to stress	
VIT_13s0019g02770	5.97	6.52	heat shock protein 16.9 kDa class I	response to stress	
VIT_17s0053g00010	5.94	2.92	no hit		↓
VIT_09s0002g00630	5.83	5.07	no hit		
VIT_07s0141g00130	5.64	5.14	protein phosphatase 2C	protein metabolism	↓
VIT_19s0014g02510	5.63	5.80	no hit		↓
VIT_06s0004g05680	5.58	6.06	Glutathione S-transferase 25 GSTU7	response to stress	↓
VIT_00s0301g00040	5.46	5.92	glycine-rich protein	metabolic process	↓
VIT_19s0015g01300	5.43	4.14	amino acid permease 7	cellular amino acid metabolism	↓
VIT_09s0070g00740	5.39	4.21	pfkB-type carbohydrate kinase	biological process	↓
VIT_19s0135g00190	5.37	5.12	CYP72A59	secondary metabolism	↓
VIT_00s2608g00010	5.32	4.71	photosystem II PsbB	photosynthesis	↓
VIT_15s0024g01960	5.31	3.66	DNA-directed RNA polymerase subunit beta'	transcription	↓
VIT_00s0246g00200	5.29	3.87	Photosystem I iron-sulfur center (Photosystem I subunit VII)	photosynthesis	↓
VIT_02s0033g00410	5.26	4.75	myb VvMYBA1	secondary metabolism	
VIT_19s0093g00310	5.14	5.93	Glutathione S-transferase 8 GSTU19	response to stress	↓
VIT_08s0040g01100	5.10	5.10	S-adenosylmethionine synthetase 1 (SAM1)	hormone metabolism	↓
VIT_14s0108g01640	5.03	3.01	NADH dehydrogenase ND2	carbohydrate metabolism	↓
VIT_16s0050g01250	5.03	6.72	copper chaperone (CCH)	transport	↓
VIT_14s0060g01170	4.97	5.09	cytochrome c oxidase subunit Vc	photosynthesis	↓
VIT_13s0064g01420	4.91	5.32	succinate dehydrogenase [ubiquinone] flavoprotein subunit	carbohydrate metabolism	↓
VIT_19s0015g02890	4.90	5.78	Glutathione S-transferase 25 GSTU25	response to stress	↓
VIT_18s0075g00560	4.88	5.11	fatty acid multifunctional protein (MFP2)	metabolic process	↓
VIT_15s0048g00140	4.77	5.11	H(+)-ATPASE AHA10	biological process	↓
VIT_09s0002g03290	4.72	5.36	peroxiredoxin (alkyl hydroperoxide reductase subunit C)	response to stress	↓
VIT_19s0015g02940	4.63	5.13	CYP72A59	secondary metabolism	↓
VIT_00s0246g00060	−5.00	−5.00	cytochrome c-type biogenesis protein CcmF	transport	↑
VIT_00s0246g00080	−8.08	−8.08	NADH dehydrogenase subunit 7	carbohydrate metabolism	↑
VIT_10s0116g01110	−11.70	−11.70	no hit		↑

The NDR genes with the greatest fold change in expression compared to C in both treatments (Table 3) predominantly represented the functional categories “Photosynthesis” and “Response to stress” (Additional file 3) indicating that defoliation delays the switch-down of photosynthetic activity and the oxidative burst that marks the onset of ripening [31]. The NDR stress-response genes included two L-ascorbate peroxidases (VIT_19s0014g02410 and VIT_17s0053g00180), a peroxiredoxin (VIT_09s0002g03290), two superoxide dismutases (VIT_14s0030g00830 and VIT_14s0030g00950) and a copper chaperone (VIT_16s0050g01250) that delivers copper to the superoxide dismutase [32], 10 glutathione S-transferases (GSTs) and several heat shock proteins.

Many other NDR genes common to both defoliation treatments (Additional file 3) were related to “Carbohydrate metabolism”, including the cytosolic isoform of malate dehydrogenase (MDH, VIT_07s0005g03360), which maintains a balance between malic acid and oxalacetic acid [33], and a phosphoenolpyruvate carboxylase (PEPC, VIT_19s0015g00410), which converts PEP to oxalacetic acid. PEPC expression is higher during early fruit development but downregulated at veraison, mirroring the profiles of malate accumulation [34,35]. These data suggest that malate synthesis is prolonged in PB and V berries compared to C, although there was no significant difference in titratable acidity until harvest (Table 2 and Additional file 1). Since we did not analyze malic acid and tartaric acid separately we can only speculate that the anticipated higher malic acid content at the onset of ripening is counterbalanced by the higher temperature of berries in defoliated vines.

The carbohydrate-related NDR genes also included two mannosyl-oligosaccharide 1,2-α-mannosidases (VIT_09s0054g00320 and VIT_09s0054g00230) potentially involved in the turnover of N-glycoproteins [36]. The downregulation of such genes during ripening has previously been reported [34] again supporting our hypothesis that defoliation delays ripening. Other NDR genes were found to be involved in sucrose and starch metabolism, including three sucrose synthases, a sucrose phosphatase, two glucose-6-phosphate translocators, a tonoplast monosaccharide transporter, a β-amylase 1 and two galactinol synthases. Still others were related to fermentative metabolism, including three alcohol dehydrogenases and two aldehyde dehydrogenases. Carbohydrate mobilization and the induction of fermentative metabolism are common features of berry ripening, which again appeared to be delayed in the PB and V berries.

Many ethylene-related and ABA-related genes were also found in the NDR group, and auxin-related genes were found in both the NDR and NUR groups, suggesting that defoliation disrupts the hormonal signals that mediate ripening. At least nine “Cell wall metabolism” genes were also found in the NDR category, including four β-tubulins and three GTPase activators. A small number of phenylpropanoid-related genes including three cinnamyl alcohol dehydrogenases and a 4-coumarate-CoA ligase were also identified, as discussed in more detail later.

Finally, a minority of genes was modulated by genuine upregulation or downregulation in the PB and V berries (Table 3 and Additional file 3). These genes probably explain the biochemical and physiological modifications in PB and V berries that do not reflect delayed ripening, and are therefore particularly interesting because they reveal the specific transcriptomic impact of defoliation. This group included several upregulated heat shock proteins (presumably reflecting the higher temperature of defoliated clusters), storage proteins such as glutelin type-A 3 (VIT_12s0034g01910) and legumin (VIT_12s0034g01950), as well as the anthocyanin regulator *VvMYBA1* (VIT_02s0033g00410) [37], a flavonoid 3'-hydroxylase (VIT_s0000g07210) and a flavonol synthase (VIT_18s0001g03470). These genes may contribute to the uncoupling of flavonoid metabolism from other ripening parameters as discussed below.

### Time-dependent impact of defoliation on the berry transcriptome

Among the 669 EV genes differentially expressed solely between PB and C berries and the 95 EV genes differentially expressed solely between V and C berries, few showed a high fold-change in expression (Additional file 2). As for the common set of genes, many of the time-dependent genes also fell into the NDR category whereas a small number were genuinely upregulated or downregulated by defoliation.

The PB-specific dataset included several NDR genes related to sugar metabolism (Additional file 4), although these excluded the major sugar transport genes involved in ripening, such as sucrose transporters and hexose transporters [38]. It also included several NDR and NUR genes related to photosynthesis and oxidative stress, suggesting that the ripening delay effect is more pronounced in PB than V berries. Other stress-related genes were genuinely upregulated specifically in PB berries, including a cold shock protein, a dehydration-response protein, two heat shock proteins, and a stress responsive αβ-barrel domain, all potentially associated with the early exposure of PB berries to sunlight.

Cell wall metabolism also appeared to be affected more strongly in PB than V berries, including the genuine downregulation of an α-expansin, a pectin methylestarase inibitor, a xyloglucan endotransglycosylase (XET) and a wax synthase, plus the genuine upregulation of a mannosidase and a cellulose synthase. These differences may account for specific cell wall features in ripe PB berries, such as skin thickness at harvest (Table 2). Several genes related to the phenylpropanoid/flavonoid pathway were specifically modulated in PB berries, as discussed later.

The V-specific dataset included genuinely upregulated stress-related genes such as an aldo/keto reductase, a class IV chitinase, a thaumatin, an early light-inducible protein and a heat shock protein, all potentially reflecting the sudden exposure to sunlight after veraison (Additional file 4). Similarly we detected the genuine upregulation of a cell wall hydrolase, an endo-1,3;1,4-β-D-glucanase precursor, a pectin methylesterase inhibitor and an XET, which may help to protect berries from sunburn.

### Gene expression profiles during ripening depend on the timing of defoliation

We integrated the expression data using a SAM multiclass comparison to identify additional transcripts differentially expressed in PB and V berries. This revealed 2470 genes modulated in C berries, 2392 genes modulated in PB berries, and 1789 genes modulated in V berries during ripening, with a fold change ≥ 2 in at least one comparison (Additional file 5). Clustering analysis using Pearson’s correlation distance divided the C, PB and V modulated transcripts into eight groups representing the minimum number of profiles required to describe gene expression along the three sampling time points. Clusters 1–4 represented genes that are downregulated during at least one time point compared to the BV stage, whereas clusters 5–8 represent genes that are upregulated during at least one time point compared to the BV stage (Additional file 6). By focusing on genes with a fold change ≥3 we identified 529 genes clustering differently in at least one condition but not present among the genes already identified by the direct comparison approach described above (Additional file 7). These genes were divided into four groups: 229 genes with like clustering in both defoliation treatments compared to C (common effect), 56 genes with distinct clustering in both defoliation treatments compared to C (different effect), 156 genes with cluster shifting only in PB berries (PB-specific effect) and 88 genes with cluster shifting only in V berries (V-specific effect) (Additional file 7). We interpreted the cluster shifts to mean either an anticipated or delayed effect compared to C berries or to mean a novel form of modulation or non-modulation (Additional file 7). This showed the number of genes with delayed modulation in PB and V berries compared to C was always greater than the number with anticipated modulation, supporting our hypothesis that the transcriptomic changes associated with ripening are generally delayed by defoliation. The genes selected by clustering included those related to “Photosynthesis”, “Carbohydrate metabolism”, “Hormone metabolism”, “Stress response” and “Phenypropanoid/Flavonoid metabolism” (Additional file 8). We focused on genes potentially related to the compositional diversity of treated berries and identified three more candidates involved in organic acid metabolism that could help to explain the lower titratable acidity in PB and V berries. Both treatments delayed the downregulation of two tonoplast dicarboxylate transporters (VIT_00s0187g00130 and VIT_00s2188g00010) potentially involved in the uptake of malate and/or tartrate to the vacuole but the downregulation of L-idonate-dehydrogenase (VIT_16s0100g0029), which is considered to be the rate-limiting step in tartaric acid synthesis [39], was only delayed in PB berries. L-idonate-dehydrogenase is strongly expressed in pre-veraison berries, mirroring tartaric acid accumulation during berry ripening. Tartaric acid levels in ripe berries reflect the extent of synthesis during early development because there is negligible degradation at later stages. Overall, considering also the above discussed downregulation of MDH and PEPC genes, the observed expression of acidity-related genes suggests a general delay in the switch-down of organic acid biosynthesis, which should increase the titratable acidity of PB and V berries*.* The higher titratable acidity previously reported in berries from defoliated vines probably reflects the increased synthesis of tartaric acid [16,40]. Hence, the inconsistence with our results showing the lower titratable acidity of the PB berries compared to C probably reflects temperature-dependent malic acid degradation.

Clustering analysis also showed that the downregulation of vacuolar invertase 1 (*GIN1,* VIT_16s022g00670) was delayed in both treatments and thus cannot explain alone the difference in soluble solids content between PB and V berries. However, this might be explained by the non upregulation of sucrose transporter 4 (*SUT4*, VIT_18s0001g08220) in V berries and its late upregulation in PB berries compared to C (Additional file 8).

### Impact of defoliation on the expression of phenylpropanoid and flavonoid pathway genes

**Figure 4:**
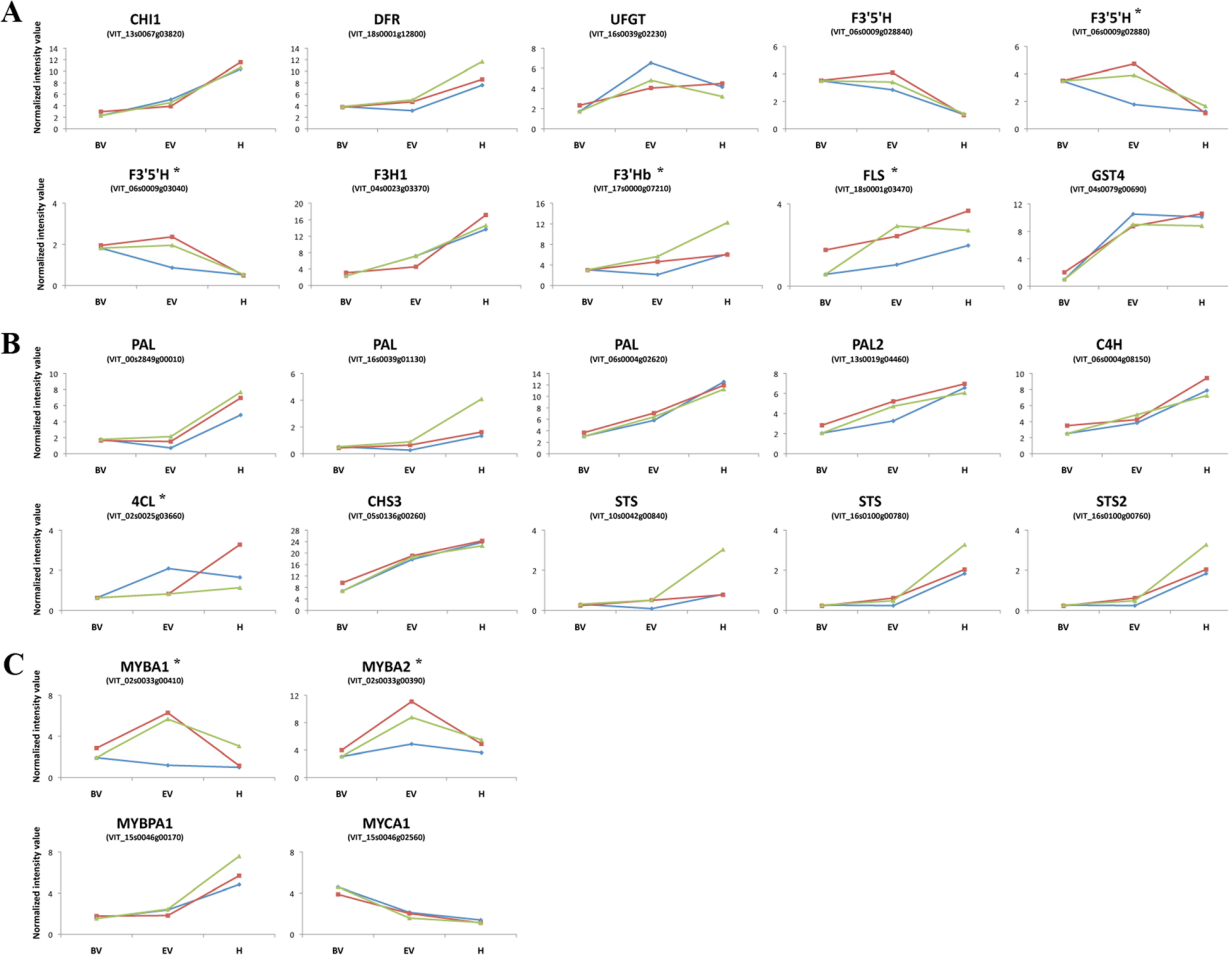
**Expression profiles of genes related to the phenylpropanoid pathway, differentially expressed in PB (red line) and V (green line) berries compared to C (blue line), resulting from either direct comparison at each time point or clustering analysis.** Genes resulted from the direct comparison are marked with “*”. (**A**) Structural flavonoid pathway genes. (**B**) Structural phenylpropanoid pathway genes. (**C**) Regulatory genes.

The impact of defoliation on phenylpropanoid/flavonoid metabolism was explored by considering the results of the two statistical approaches described above, i.e. the direct comparison of time course stages and the clustering analysis of expression profiles. We selected all genes related to phenylpropanoid/flavonoid metabolism either differentially expressed with a FC ≥ 2 between C and PB or V at each time point (Additional file 2), or showing different cluster of expression profile between C and the two treatments (Additional file 7). A total of 24 phenylpropanoid/flavonoid-related genes resulted differentially modulated in PB and V berries compared to C by one or the other statistical approach (Figure 4). Although in some cases the differences in expression of these genes in C, PB and V berries appeared very small, and of questionable biological significance when considered alone, a general overview on the expression level of these phenylpropanoid/flavonoid pathway genes allowed us to highlight some apparent features characterizing each treatment as discussed below. The accumulation of total flavonols in PB and V berries compared to C (Table 1, Figure 2A) is supported by the increase in FLS expression in PB berries at the BV stage and in V berries following defoliation (Figure 4A). The lower anthocyanin content of V berry skins compared to C (Table 1, Figure 2B) is supported by the reduction in UDP glucose:flavonoid-3-O-glucosyltransferase (UFGT) and GST4 expression, which are directly involved in anthocyanin synthesis and transport. However this does not hold true for PB berries, where the marginally higher anthocyanin content compared to C does not reflect the expression pattern of UFGT and GST4, which are expressed at lower levels compared to C berries at the EV stage (Figure 4A). Genes acting upstream of UFGT in the biosynthesis pathway may provide an explanation, e.g. the marginally higher expression of dihydroflavonol reductase (DFR) may increase the flow of substrates towards anthocyanins in PB berries (Figure 4A).

F3^′^Hb (flavonoid 3^′^ hydroxylase) and F3^′^5^′^H (flavonoid 3^′^5^′^ hydroxylase) could also help to channel substrates into the anthocyanin branch. Mono-substituted anthocyanins (e.g. pelargonidin derivatives) are not usually synthesized from dihydrokaempferol in grapevine, but dihydrokaempferol is converted by F3^′^Hb into dihydroquercetin and by F3^′^5^′^H into dihydromyricetin, which are used as substrates by DFR for anthocyanin synthesis [41]. F3^′^Hb and three F3^′^5^′^Hs are expressed at high levels at EV in PB berries compared to C (Figure 4A).

The differential expression of genes from the early steps of the phenylpropanoid pathway could also influence anthocyanin levels in berry skins after defoliation (Figure 4B). The induction of stilbene synthase (STS) genes during late ripening has been reported in other cultivars [42-44], and we noted the stronger upregulation of three STS genes in V compared to PB and C berries at harvest (Figure 4B). STS and chalcone synthase (CHS) compete for the same substrates to synthesize stilbenes and flavonoids, respectively. Therefore, the higher expression of STS genes in V berries could divert flux from the anthocyanin pathway to the stilbene pathway.

The relatively low proportion of tri-substituted anthocyanins (mainly malvidin) found in V berries is supported by the higher expression of a F3^′^Hb [45]. This gene is only marginally induced in PB berries compared to C at the BV and EV stages. The concomitant up regulation of F3^′^5^′^H may counterbalance the activity associated with F3^′^Hb (Figure 4A), resulting in the similar anthocyanin profile is in PB and C berries (Table 1).

We also identified three Myb transcription factors that were differentially expressed in one or both of the defoliation treatments, namely MYBA1 (VIT_02s0033g00410), MYBA2 (VIT_02s0033g00390) and MYBPA1 (VIT_15s0046g00170) (Figure 4C). MYBA1 and MYBA2 are known to control the expression of UFGT and GST4 in grapevine [37,46] and act as master regulators of anthocyanin biosynthesis [47]. However, the expression profiles of MYBA1 and MYBA2, validated by real time RT-PCR (Additional file 9), did not match those of UFGT and GST4 in our experiments, and were instead similar to the expression profiles of the three F3^′^5^′^H genes we identified (Figure 4A and 4C). This suggests that other regulators may influence the expression of UFGT and GST and that F3^′^5^′^H genes may be additional targets for MYBAs. Another regulator of anthocyanin biosynthesis which was differentially expressed in response to defoliation was the basic helix-loop-helix transcription factor MYCA1 (VIT_15s0045g022560) [48]. This was slightly downregulated in V compared to C berries suggesting that the higher temperature of the V berries may have reduced MYCA1 expression (Figure 4C). Apple (*Malus domestica*) MdbHLH3, which interacts with MYB transcription factors to regulate anthocyanin biosynthesis, is induced transcriptionally and modified post-translationally by cold treatment thus promoting anthocyanin accumulation in apple skins [49].

MYBPA1, which controls the expression of proanthocyanidin pathway genes [50], was found to be upregulated in all samples at harvest compared to the previous time-points, most intensely in V berries. Four flavonoid pathway structural genes showed expression profiles matching that of MYBPA1: chalcone isomerase (CHI), F3H, F3^′^Hb and DFR, with the last two confirmed by real time RT-PCR (Additional file 9). Because proanthocyanidin biosynthesis is thought to be restricted to the herbaceous phase of berry development, MYBPA1 may promote anthocyanin biosynthesis during ripening by controlling the expression of the structural genes acting upstream of UFGT, as previously proposed by Falginella et al. (2012) [47]. This would require a specific regulatory mechanism preventing the activation of leucoanthocyanidin reductase (LAR) and anthocyanidin reductase (ANR) by MYBPA1 during ripening, since these genes are also controlled by MYBPA1 and are directly involved in proanthocyanidin synthesis during early berry development. The higher level of MYBPA1 expression in V berries compared to C at harvest may reflect the positive impact of higher temperature or sunlight exposure during ripening, which contrasts with similar experiments on Cabernet Sauvignon berries [6], suggesting cultivar-dependent factors may influence the expression of MYBPA1 following defoliation. By dissecting the influence of light and temperature, Azuma et al. (2012) [51] showed that strong illumination but not high temperatures can positively affect MYBPA1 expression in detached berries. Thus the upregulation of MYBPA1 in V berries at harvest may reflect the impact of illumination rather than temperature.

## Conclusions

We compared the agronomic, biochemical and transcriptomic parameters of ripening Sangiovese berries sampled from vines defoliated before flowering or at veraison (and from untreated control vines) to determine the common and specific effects of each defoliation treatment. Both treatments influenced fruit ripening parameters and hence enological berry traits. Defoliation before flowering caused a slight increase in sugar and anthocyanin levels, whereas defoliation at veraison reduced the anthocyanin content and increased the negative impact of sunburn. Transcriptomic analysis revealed significant transcriptional changes at the end of veraison in the berries from defoliated vines. Different analytical approaches (PCA, direct comparison at each time point, and the analysis of gene expression profiles) indicated a general delay of the transcriptional ripening program following both defoliation treatments, but also common and time-dependent defoliation effects uncoupled from the general ripening program, which was particularly apparent for many structural and regulatory genes controlling anthocyanin biosynthesis. The specific transcriptional modifications we observed following defoliation at different time points allow the identification of the developmental or metabolic processes affected in berries thus deepening the knowledge of the mechanisms by which these agronomical practices impact the final berry ripening traits.

## Methods

### Plant material

We tested adult *Vitis vinifera* L. cv Sangiovese vines (clone 12 T grafted to SO4, 2008 vintage) in a non-irrigated vineyard in Bologna, Italy (44°30^′^N, 11°24^′^E), with north–south oriented rows. The vines, spaced 1.0 m within the row and 2.8 m between rows, were trained to a vertical-shoot-positioned spur-pruned cordon (12 buds per vine) with a cordon height 1.0 m above ground and a canopy wall of 1.3–1.4 m over the cordon. Hedging was performed on all vines on Julian Day (JD) 192, when most had started to outgrow the top wire, and pest management was carried out according to Regione Emilia Romagna local practice. Nine vines per treatment, with the same cluster number at flowering (16 per vine), were selected in a single uniform row, and each vine was randomly assigned to three blocks, each of them representing three treatments: (i) control (C) with no defoliation; (ii) pre-bloom manual defoliation (PB) of the main and lateral leaves in the first six basal shoot nodes at stage 17 (JD 147, inflorescence fully developed and single flowers separated); and (iii) veraison manual defoliation (V) as above at veraison (JD 211, berries softening and °Brix ~8). Stages were defined according to Eichorn and Lorenz (1977) [52].

### Leaf area measurements

The main and lateral leaf areas removed in the PB and V treatment groups were measured separately with a LI-3000A leaf area meter (Li-Cor Biosciences, Lincoln, Nebraska, USA). The main and lateral shoot lengths and the corresponding leaf areas were measured in 15 shoot samples from each treatment group before defoliation and at harvest. The resulting regressions (data not shown) were then used to calculate the total main and lateral leaf areas of each vine based on the length of all shoots and their individual laterals.

### Berry skin temperature measurements

Berry skin temperature was monitored in two clusters from each treatment group (representing the east and west sides of each row, respectively) using 24 T-type thermocouples (RS components, MI, Italy) positioned in the subcuticular tissues of the berry skin. Two thermocouples were placed externally and two internally in each cluster. Each probe was connected to a CR10X data logger (Campbell Scientific Ltd., Leicestershire, United Kingdom), registering temperature data every 15 min from stage 33 (JD 192, bunch closure) to harvest (JD 266).

### Biochemical analysis

For each treatment, we collected 40 berries from three vines in each block at the following stages [52]: (i) JD 182, pre-bunch closure, berries touching; (ii) beginning of veraison (JD 211, berries softening, °Brix ~8); (iii) full veraison (JD 219); (iv) end of veraison (JD 227, soft and fully-colored berries); (v) ripening (JD 250); and (vi) ripe (JD 266, harvest). The samples were divided into two parts. Twenty berries were weighed and immediately tested for ripening by crushing and filtering the must through a strainer for the evaluation of °Brix, titratable acidity and pH [20]. The remaining 20 berries were used to extract anthocyanins and flavonols for HPLC analysis [20,29].

### Morphology and agronomic parameters at harvest

We collected 100 berries from each vine per treatment group and measured skin thickness using a Universal Testing Machine (UTM) TAxT2j Texture Analyzer (Stable Micro Systems Surrey, UK) as described by Letaief et al. (2008) [53]. We measured the yield, cluster number and cluster weight, and for each cluster we determined the surface area infected by bunch rot, the surface area damaged by sunburn, and the index of cluster compactness (according to the 1983 OIV classification). We also counted the number of berries in two clusters picked from each vine.

### Microarray analysis

Thirty additional berries collected randomly from three vines in each block per treatment at beginning of veraison (BV), end of veraison (EV) and full ripening (H) were immediately frozen in liquid nitrogen and stored at −80°C. Each pool of thirty berries from a block represented a biological replicate. Total berry pericarp RNA was extracted, quantified and tested for integrity as previously described [20]. The RNA was hybridized to a NimbleGen microarray 090818 Vitis exp HX12 (Roche, NimbleGen Inc., Madison, WI) containing probes for 29,549 grapevine genes based on the 12X grapevine V1 gene prediction (http://genomes.cribi.unipd.it/grape/index.html). All microarray expression data are available in the GEO under the series entry GSE40487 (http://www.ncbi.nlm.nih.gov/geo/query/acc.cgi?acc=GSE40487).

Pearson correlation analysis and principal component analysis (PCA) were carried out using SIMCA P+ (Umetrics, Umea, Sweden) to evaluate the robustness of the three biological replicates in each treatment per stage. A gene was designated as expressed if the normalized expression value was higher than the value obtained by averaging the fluorescence of the negative control present on the chip, for at least two of the three biological replicates. A Significance Analysis of Microarrays (SAM) approach was implemented using TMeV software (http://www.tm4.org/mev) with a false discovery rate of 2%. We also carried out k-means clustering using Pearson’s correlation distance using TMeV to compare gene expression in the three developmental stages.

### Real-time RT-PCR

We prepared cDNA from the total RNA extracted for microarray analysis, followed by DNase treatment and amplification by PCR as described by Pastore et al. (2011) [20]. Gene-specific primers were designed for four phenylpropanoid pathway-related genes using the sequence information in the 3^′^-UTR or in specific coding regions (Additional file 10), and the elongation factor 1 (*EF1*) gene was used as reference [54]. Amplification efficiency was calculated from raw data using LingRegPCR software [55]. The relative expression ratio was calculated relative to the first sampling time point (BV) according to the Pfaffl equation [56]. Standard error (SE) values were calculated according to Pfaffl et al. (2002) [57]. Final data were calculated as previously reported [58].

## Competing interests

The authors declare that they have no competing interests.

## Authors’ contributions

CP carried out the biochemical and transcriptomic studies and drafted the manuscript. SZ performed the statistical analyses and drafted the manuscript. MF participated in the transcriptomic analyses and performed Real Time RT-PCR. MP participated in the study design and coordination. GBT performed the statistical analyses and wrote the manuscript. IF conceived the study and helped to draft the manuscript. All authors read and approved the manuscript.

## Supplementary Material

Additional file 1 Effects of pre-bloom (PB) and veraison (V) defoliation compared to an untreated control (C) on Sangiovese berry weight (A), soluble solids (B) and titratable acidity (C). Vertical bars indicate the standard deviation of the mean (n = 3 biological replicates). Click here for file

Additional file 2 **Differentially-expressed genes displaying a two-fold or greater difference in transcript abundance between C and PB berries at EV and H, and between C and V berries at EV.** For each gene, we show the PB/C and V/C fold-change (FC), the description, the GO functional category and the increase (↑) or decrease (↓) in expression between BV and EV in C berries. Click here for file

Additional file 3 **Common differentially-expressed genes between C and PB and C and V berries at EV, displaying a two-fold or greater change in transcript abundance and belonging to selected functional categories.** For each gene, we show the fold-change (FC) at EV, the description and the increase (↑) or decrease (↓) in expression between BV and EV in C berries. Click here for file

Additional file 4 **Specific differentially-expressed genes between C and PB and C and V berries at EV, displaying a two-fold or greater change in transcript abundance and belonging to selected functional categories.** For each gene, we show the fold-change (FC) at EV, the description and the increase (↑) or decrease (↓) in expression between BV and EV in C berries. Click here for file

Additional file 5 **Differentially-expressed genes during berry development displaying a two-fold or greater change in transcript abundance between EV and BV or EV and H, in C, PB and V berries.** For each gene, we show the description, the EV/BV and H/BV fold-change (FC) and the cluster number. Data obtained for C, PB and V berries are listed in three separate worksheets. Click here for file

Additional file 6 Representative expression profiles of the eight clusters. Click here for file

Additional file 7 **Differentially-expressed genes during berry development displaying a three-fold or greater change in transcript abundance between EV and BV or EV and H, in C, PB and V berries.** For each gene, we show the description, the EV/BV and H/BV fold-change (FC), the cluster number or the non-modulation (NM) in each treatment, and the effect on gene expression in comparison to C berries. Genes commonly affected in the PB and V berries, genes differentially affected in the PB and V berries, genes specifically affected in PB berries and genes specifically affected in V berries are listed in four separate worksheets. Click here for file

Additional file 8 **Genes belonging to selected functional categories, differentially-expressed during berry development, displaying a three-fold or greater change in transcript abundance between EV and BV or EV and H, in C, PB and V berries.** For each gene, we show the description and the effect on gene expression in comparison to C berries. Downregulation, downregulation advanced, upregulation delayed and no upregulation effects compared to C berries are indicated in green; upregulation, upregulation advanced, downregulation delayed and no down regulation effects compared to C berries are indicated in red; gray color indicates no difference compared to C. Click here for file

Additional file 9 **Real time RT-PCR validation of MYBA1/A2 (VIT_02s0033g00410 and VIT_02s0033g00390), F3**^**′**^**Hb (VIT_17s0000g07210), DFR (VIT_18s0001g12800), and MYBPA1 (VIT_15s0046g00170) expression profiles in pre-bloom defoliated (PB), veraison defoliated (V) and control (C) berries during ripening.** The amplification of MYBA1 and MYBA2 transcripts was performed using a primer pair that recognizes both sequences [47]. Expression profiles measured by real time RT-PCR were determined by calculating the relative expression *ratio* value for each stage relative to the BV stage. Real time RT-PCR data are reported as means ± SE of three biological replicates, obtained using elongation factor 1 (VIT_06s0004g03220) for normalization. Click here for file

Additional file 10 List of the primers used for real time RT-PCR. Click here for file
