# Supplementary material for: Selective defoliation affects plant growth, fruit transcriptional ripening program and flavonoid metabolism in grapevine
Source: BMC Plant Biol. 2013 Feb 22;13:30. doi: 10.1186/1471-2229-13-30 (PMC3599245; doi:10.1186/1471-2229-13-30)

Additional file 1. Effects of pre-bloom (PB) and veraison (V) defoliation compared to an untreated control (C) on Sangiovese berry weight (A), soluble solids (B) and titratable acidity (C). Vertical bars indicate the standard deviation of the mean (n = 3 biological replicates).

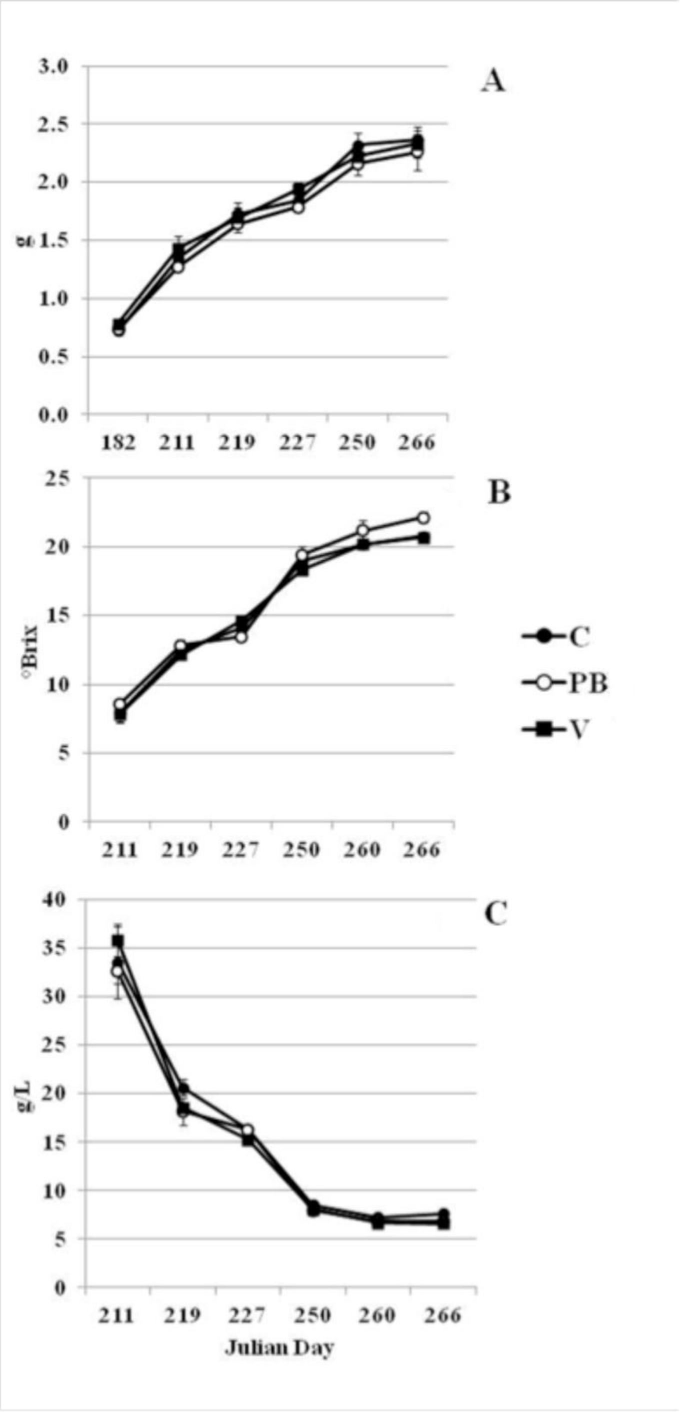

Supplement: Additional file 1 — Effects of pre-bloom (PB) and veraison (V) defoliation compared to an untreated control (C) on Sangiovese berry weight (A), soluble solids (B) and titratable acidity (C). Vertical bars indicate the standard deviation of the mean (n = 3 biological replicates). [file 1471-2229-13-30-S1.pdf]
