# Supplementary figures and images for: Selective defoliation affects plant growth, fruit transcriptional ripening program and flavonoid metabolism in grapevine
Source: BMC Plant Biol. 2013 Feb 22;13:30. doi: 10.1186/1471-2229-13-30 (PMC3599245; doi:10.1186/1471-2229-13-30)

Additional file 6

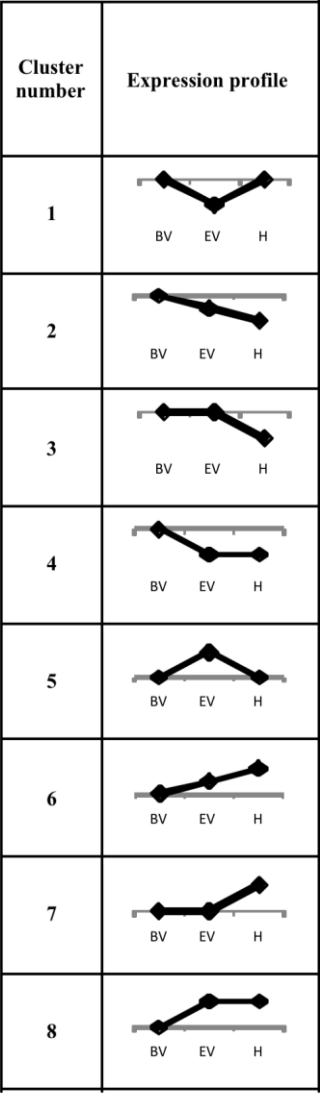

Supplement: Additional file 6 — Representative expression profiles of the eight clusters. [file 1471-2229-13-30-S6.pdf]
