# Supplementary material for: Selective defoliation affects plant growth, fruit transcriptional ripening program and flavonoid metabolism in grapevine
Source: BMC Plant Biol. 2013 Feb 22;13:30. doi: 10.1186/1471-2229-13-30 (PMC3599245; doi:10.1186/1471-2229-13-30)

Additional file 9. Real time RT-PCR validation of MYBA1/MYBA2 (VIT_02s0033g00410/ VIT_02s0033g00390), F3'Hb (VIT_17s0000g07210), DFR (VIT_18s0001g12800), and MYBPA1 (VIT_15s0046g00170) expression profiles in pre-bloom defoliated (PB), veraison defoliated (V) and control (C) berries during ripening. The amplification of MYBA1 and MYBA2 transcripts was performed by using a primer pair that recognizes both sequences [47]. Expression profiles measured by real time RT-PCR were determined by calculating the relative expression *ratio* value for each stage relative to the BV stage. Real time RT-PCR data are reported as means ± SE of three biological replicates, obtained using elongation factor 1 (VIT_06s0004g03220) for normalization.

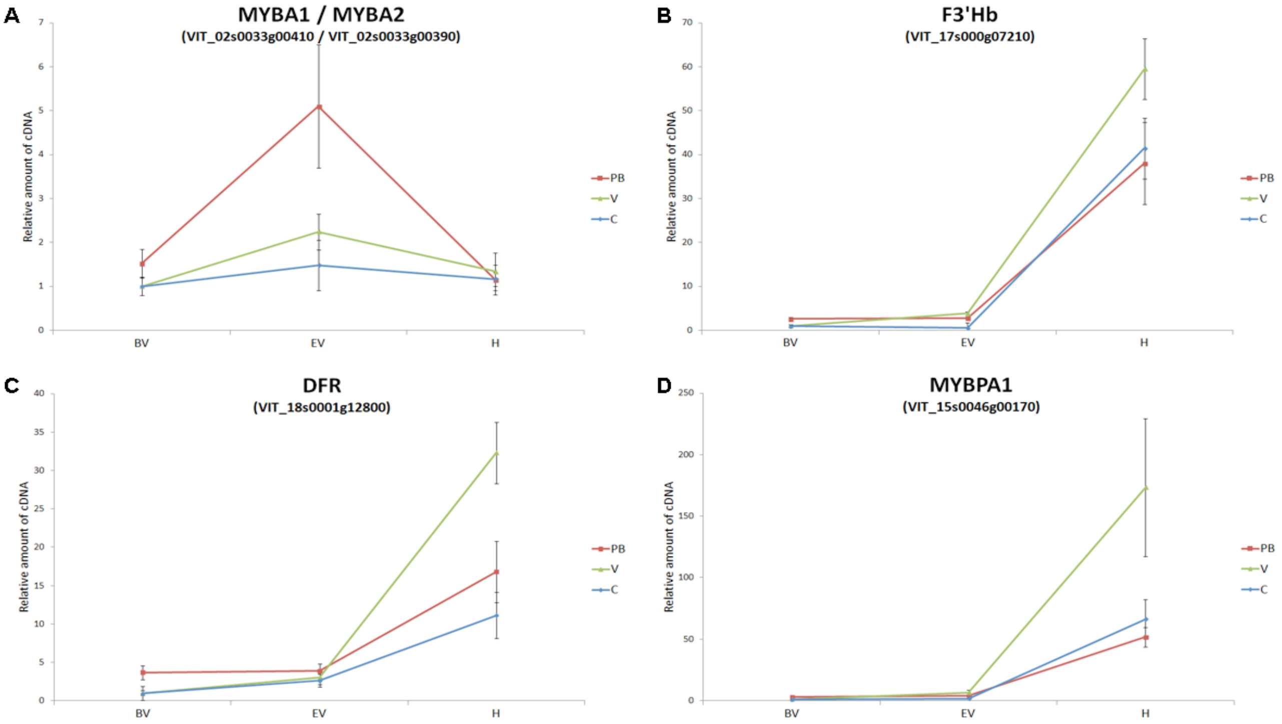

Supplement: Additional file 9 — Real time RT-PCR validation of MYBA1/A2 (VIT_02s0033g00410 and VIT_02s0033g00390), F3′Hb (VIT_17s0000g07210), DFR (VIT_18s0001g12800), and MYBPA1 (VIT_15s0046g00170) expression profiles in pre-bloom defoliated (PB), veraison defoliated (V) and control (C) berries during ripening. The amplification of MYBA1 and MYBA2 transcripts was performed using a primer pair that recognizes both sequences [47]. Expression profiles measured by real time RT-PCR were determined by calculating the relative expression ratio value for each stage relative to the BV stage. Real time RT-PCR data are reported as means ± SE of three biological replicates, obtained using elongation factor 1 (VIT_06s0004g03220) for normalization. [file 1471-2229-13-30-S9.pdf]
