# Supplementary material for: Selective defoliation affects plant growth, fruit transcriptional ripening program and flavonoid metabolism in grapevine
Source: BMC Plant Biol. 2013 Feb 22;13:30. doi: 10.1186/1471-2229-13-30 (PMC3599245; doi:10.1186/1471-2229-13-30)
Supplement: Additional file 10 — List of the primers used for real time RT-PCR. [file 1471-2229-13-30-S10.pdf]

Additional file 10. List of the primers used for real time RT-PCR.

Gene-ID	*Forward primer*	*Reverse primer*
VIT_02s0033g00410/ VIT_02s0033g00390	5'-GAGGGTGATTTTCCATTTGAT-3'	5'-CAAGAACAACTTTTGAACTTAAACAT-3'
VIT_17s0000g07210	5'-TCGGGAAGTAAATGAGGTTGT-3'	5'-TCACAAAACTACTACACTCGC-3'
VIT_18s0001g12800	5'-ACCTGTAGATGGCAAGACCT-3'	5'-AAATCAAACTACCAGAAAACCTT-3'
VIT_15s0046g00170	5'-AAGGTTATTGGGGTTGACGA-3'	5'-AGTTTGTCCAGAGAGTTGTCA-3'
VIT_06s0004g03220	5'-GAACTGGGTGCTTGATAGGC-3'	5'-AACCAAAATATCCGGAGTAAAAGA-3'
